# Mitigation of Prion Infectivity and Conversion Capacity by a Simulated Natural Process—Repeated Cycles of Drying and Wetting

**DOI:** 10.1371/journal.ppat.1004638

**Published:** 2015-02-09

**Authors:** Qi Yuan, Thomas Eckland, Glenn Telling, Jason Bartz, Shannon Bartelt-Hunt

**Affiliations:** 1 Department of Civil Engineering, Peter Kiewit Institute, University of Nebraska-Lincoln, Omaha, Nebraska, United States of America; 2 Department of Medical Microbiology and Immunology, Creighton University, Omaha, Nebraska, United States of America; 3 Department of Microbiology, Immunology and Pathology, Prion Research Center, Colorado State University, Fort Collins, Colorado, United States of America; Dartmouth Medical School, UNITED STATES

## Abstract

Prions enter the environment from infected hosts, bind to a wide range of soil and soil minerals, and remain highly infectious. Environmental sources of prions almost certainly contribute to the transmission of chronic wasting disease in cervids and scrapie in sheep and goats. While much is known about the introduction of prions into the environment and their interaction with soil, relatively little is known about prion degradation and inactivation by natural environmental processes. In this study, we examined the effect of repeated cycles of drying and wetting on prion fitness and determined that 10 cycles of repeated drying and wetting could reduce PrP^Sc^ abundance, PMCA amplification efficiency and extend the incubation period of disease. Importantly, prions bound to soil were more susceptible to inactivation by repeated cycles of drying and wetting compared to unbound prions, a result which may be due to conformational changes in soil-bound PrP^Sc^ or consolidation of the bonding between PrP^Sc^ and soil. This novel finding demonstrates that naturally-occurring environmental process can degrade prions.

## Author Summary

Prion diseases such as chronic wasting disease and scrapie are emerging in North America at an increasing rate. Infectious prions are introduced into the environment from both living and dead animals where they can bind to soil. Little information is available on the effect of prion inactivation under conditions that would be found in the natural environment. In this study, we exposed both unbound and soil-bound prions to repeated cycles of drying and wetting to simulate ambient environmental conditions. We found evidence of prion inactivation in both unbound and soil bound prions. The influence of repeated cycles of drying and wetting are dependent on the prion strain and soil type used and, interestingly, prions bound to soil were more susceptible to inactivation. This is the first report of natural environmental processes mitigating prion infectivity. This data suggests that the total environmental prion load is a balance between input and natural clearance.

## Introduction

Prion diseases, also known as transmissible spongiform encephalopathies (TSEs), are a group of fatal neurodegenerative diseases which impact a number of species including cattle (bovine spongiform encephalopathy, BSE), sheep and goats (scrapie), deer, elk and moose (chronic wasting disease, CWD), and humans (Creutzfeldt-Jakob disease, CJD, and others) [1]. The infectious agent of prion diseases, PrP^Sc^, is a misfolded isoform of a non-infectious cellular prion protein, PrP^C^. CWD and scrapie prions can remain infectious over long time periods [2–5] in the environment. The increasing incidence and geographic range of CWD in cervids and its unknown host range makes this disease of particular concern in North America.

Prions enter the environment from infected hosts. Prions are shed into the environment via antler velvet [6], blood, saliva [7], urine [8–10], feces [11, 12], and birthing matter [13]. Prions also enter the environment through decomposition of infected animal carcasses [5]. Prions can be present in these excreta during the presymptomatic phase of disease, therefore, infected animals can shed prions into the environment over wide areas of the host’s home range. The amount of infectivity that is introduced into the environment is difficult to assess since prion titer is operationally defined with the route of infection, the age of the animal, the number of doses, and the PrP genotype of the host all making significant contributions in establishment of infection [14–17].

After release from an infected host, PrP^Sc^ binds to a wide range of soil and soil minerals [18–20]. Clay and clay soils have higher affinity for prions and adsorb PrP^Sc^ at a faster rate compared to sand or sandy soils [18, 19]. Binding of PrP^Sc^ to soil in a competitive matrix such as brain homogenate is slow and reduced compared to non-competitive environments (i.e. purified PrP^Sc^) [18, 19]. Once bound to soil, prions remain highly infectious although soil-induced changes in *in vitro* PrP^Sc^ conversion efficiency and infectivity in animals have been measured [21, 22]. Further work is needed to fully elucidate the effect of PrP^Sc^ binding to surfaces on prion infectivity and transmission.

The contribution of soil bound prions to the natural transmission of prion disease is incompletely understood. Modeling studies conducted in CWD-endemic areas indicate that locations with a preponderance of organic soils correspond with an increased incidence of CWD [23–25]. Since the soil type that is bound to PrP^Sc^ has a relatively small influence on prion infectivity, other factors may play a role in prion transmission. Prions are primarily retained in surface soils [26] and the close contact of ruminant animals with soils renders soil-bound prions a likely source for prion disease transmission through ingestion or inhalation [27–30]. Therefore, PrP^Sc^ binding to soil may increase the bioavailability of prions for transmission. Inactivation of soil-bound prions will be required to control and prevent the spread of prion diseases in the environment.

Prion degradation under environmentally-relevant conditions is poorly understood. To date, the majority of studies have investigated degradation and inactivation of prions that are not bound to soil. Microorganisms and isolated enzymes, sometimes associated with harsh digestion conditions (high temperature and extreme pH), effectively reduce PrP^Sc^ abundance [31–34]. Exposure of prions to intact lichens at room temperature and neutral pH can reduce the abundance of PrP^Sc^ [35]. The loss of PrP^Sc^ immunoreactivity does not always correspond with a measurable reduction of prion infectivity [33, 36] therefore, studies that rely solely on changes in PrP^Sc^ abundance must be interpreted with caution. Prionzyme, a commercially-available enzyme, degraded soil-adsorbed prions under environmentally-relevant conditions and is the first evidence to suggest that mitigation of soil-bound prions is possible [37, 38].

Prions retained in surface soils are exposed to ambient environmental processes that have the potential to inactivate prions. Naturally-occurring cycles of drying and wetting alter soil aggregate stability and can influence interactions between soil particulate organic matter and dissolved organic compounds [39–41]. They can also change microbial activity and population dynamics [39, 40, 42]. Additionally, dehydration can unfold the native protein structure [43]. It is not known if these processes alter the biologic properties of soil-bound prions. To address this important question, we investigated the effects of repeated cycles of drying and wetting on the fitness of prions bound to various soil types.

## Results

### Repeated cycles of drying and wetting reduced the abundance of total protein in hamster brain homogenate

**Fig 1:**
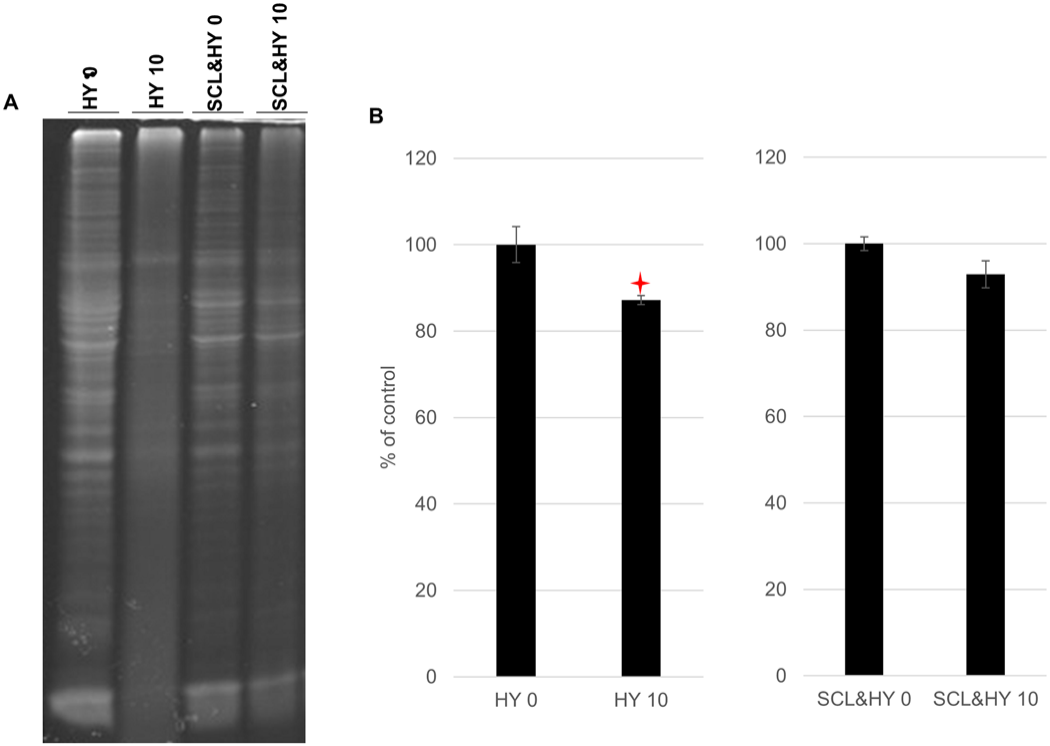
Reduced total protein abundance in HY TME brain homogenate. UV scanned polyacrylamide gel stained with SYPRO Ruby (A) and quantification of total proteins (B). Samples were not digested with proteinase K. Star indicates significant difference (p<0.05; n = 3) between treated and untreated sample.

The abundance of total protein in the brain homogenate (BH) of hamster infected with HY TME before or after binding to silty clay loam (SCL) was shown in Fig. 1. The amount of total protein of unadsorbed BH was significantly reduced (p<0.05) by 13% (Fig. 1). After binding to SCL, the amount of total protein remained unchanged (Fig. 1) suggesting protection of proteins from degradation of repeated drying and wetting by adsorption to soil surface. Repeated cycles of wetting and drying did not result in changes in pH or conductivity.

### Repeated cycles of drying and wetting alter the resistance of HY PrP^Sc^ to digestion with proteinase K

**Fig 2:**
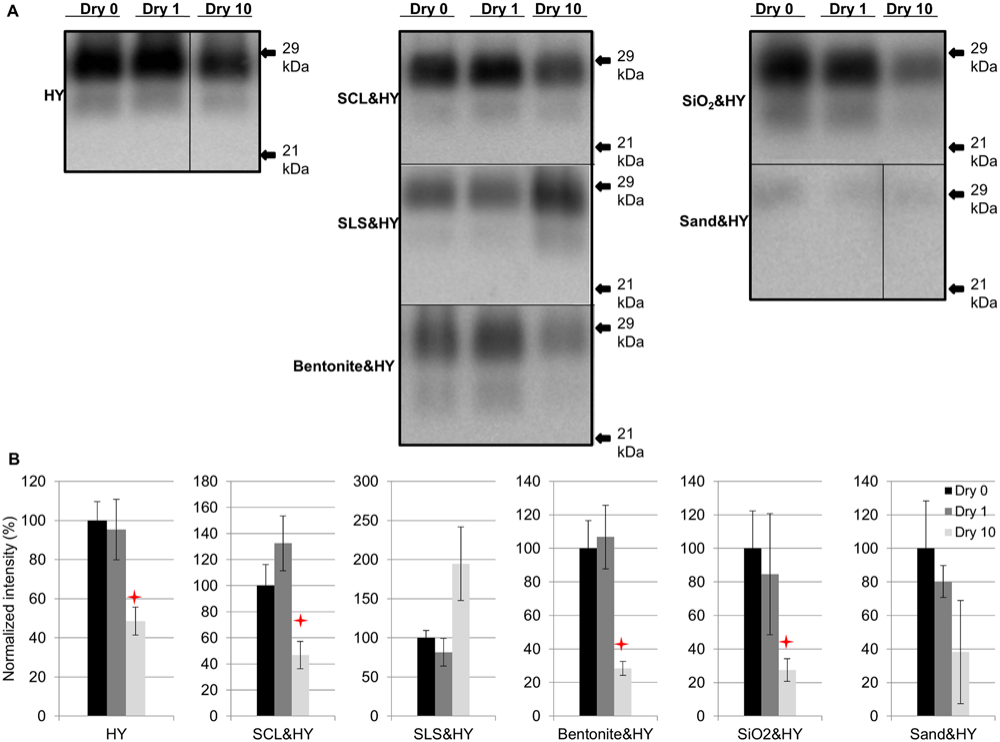
Repeated cycles of drying and wetting alter the resistance of HY PrP^Sc^ to digestion with proteinase K. Western blot (A) and quantification (B) of PK digested HY PrP^Sc^ alone or adsorbed to soil before (Dry 0) and after 1 (Dry 1) and 10 (Dry 10) serial rounds of drying and wetting. Migration of 29 and 21 kDa molecular weight marker is indicated on the right of the Western blot. Star indicates significant difference (p<0.05; n = 3) between treated and untreated sample.

Soil- or soil mineral-adsorbed HY PrP^Sc^ (HY) was prepared as described in Table 1 and subjected to 0 (control), 1, and 10 repeated cycles of drying and wetting. Significant differences (p>0.05) were not observed in normalized PrP^Sc^ immunoreactivity between samples after 1 drying and wetting cycle (Dry 1) compared to the negative control (no drying and wetting treatment, Dry 0) (Fig. 2). PrP^Sc^ abundance was significantly decreased (p<0.05) between the negative control and 10 repeated cycles of drying and wetting of unbound HY, silty clay loam (SCL)-, bentonite-, and silicon dioxide (SiO_2_)-adsorbed HY (Fig. 2). The average reductions are 51%, 53%, 72%, and 73%, respectively (Fig. 2B). The PrP^Sc^ abundance of sandy loam soil (SLS)-adsorbed and sand-adsorbed HY PrP^Sc^ were not significantly (p>0.05) changed following 10 repeated cycles of drying and wetting (Fig. 2).

**Table 1:** Preparation of soil bound HY TME, DY TME^a^, and elk CWD^a^ PrP^Sc^.

**Soil/ soil mineral**	**Incubation time (h)**	**Soil conc. (mg soil/ml solution)**	**% BH** ^b^ **(v/v)**	**Prion to soil ratio (µl BH^b^: mg soil)**	**Soil resuspension conc. (mg/µl)**	**PMCA seed^d^ (mg—µl)**
Rinda silty clay loam (SCL)	24	5	5	10	0.1	0.1–1
Bentonite clay	24	5	5	10	0.1	0.1–1
SiO_2_ powder	24	50	2.5	0.5	0.5	2–4
SiO_2-_HA^c^	168	5	5	10	0.1	1^e^
Dickinson sandy loam (SLS)	168	15	5	3.3	0.3	3^e^
Fine white sand	168	50	5	1	1	10^e^

^a^ SCL bound DY TME and elk CWD PrP^Sc^ were prepared with the same soil to brain ratio and in the same condition as SCL bound HY TME. HY TME was also used for animal bioassay

^b^ BH, 10% brain homogenate.

^c^ SiO_2-_HA, SiO_2-_humic acid.

^d^ The amount of samples used for PMCA was expressed as weight of soil—equivalent volume of soil solution.

^e^ Due to the performance challenge, PMCA substrate was added to the soil pellets of weight as indicated in the table.

### Repeated cycles of drying and wetting reduced the PMCA conversion efficiency of soil bound HY TME

**Fig 3:**
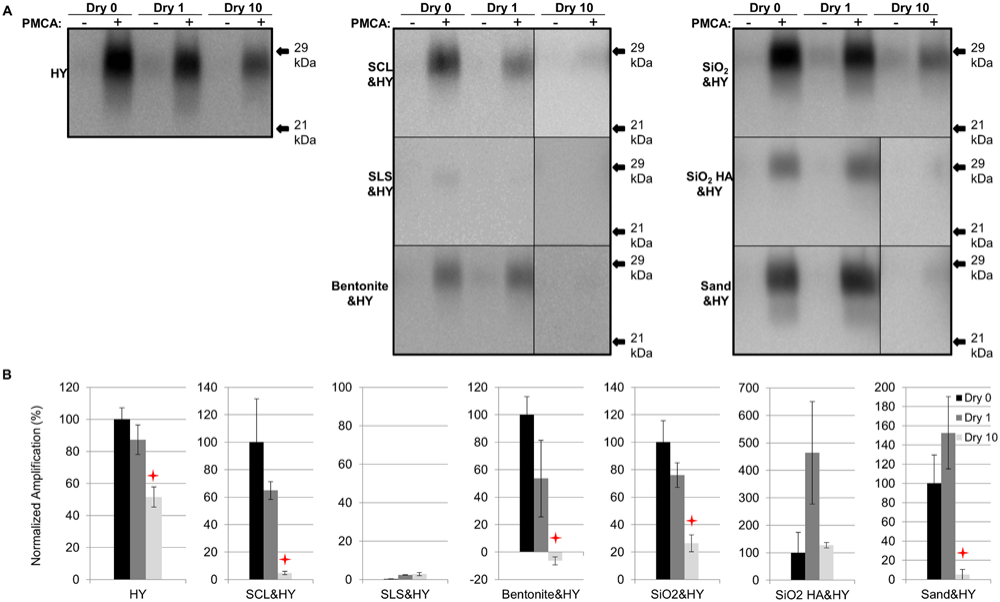
Repeated cycles of drying and wetting reduced the PMCA conversion efficiency of soil bound HY TME. Western blot (A) and quantification (B) of PMCA amplification (1 round) of HY PrP^Sc^ alone or adsorbed to soil before (Dry 0) and after 1 (Dry 1) and 10 (Dry 10) serial rounds of drying and wetting. Migration of 29 and 21 kDa molecular weight marker is indicated on the right of the Western blot. Star indicates significant difference (p<0.05; n = 3) between treated and untreated sample.

After 1 round of PMCA, PrP^Sc^ amplification in all samples subjected to 1 cycle of drying and wetting was not significantly (p>0.05) different compared to unbound HY (Fig. 3). The amplification of unbound HY, SCL-, SiO_2-_, bentonite-, and sand-adsorbed HY subjected to 10 drying/wetting cycles was significantly (p<0.05) reduced by 48%, 95%, 100% (negative value for bentonite-HY was corrected to 0%), 74%, and 95%, respectively (Fig. 3). However, the PMCA conversion efficiency of SiO_2_ HA-adsorbed HY was not changed (p>0.05) after 10 cycles of drying and wetting (Fig. 3). Sandy loam soil inhibits HY PrP^Sc^ PMCA conversion independent of HY adsorption resulting in low PrP^Sc^ abundance (Fig. 3).

### Influence of repeated cycles of drying and wetting on proteinase K resistance and amplification efficiency of DY TME

**Fig 4:**
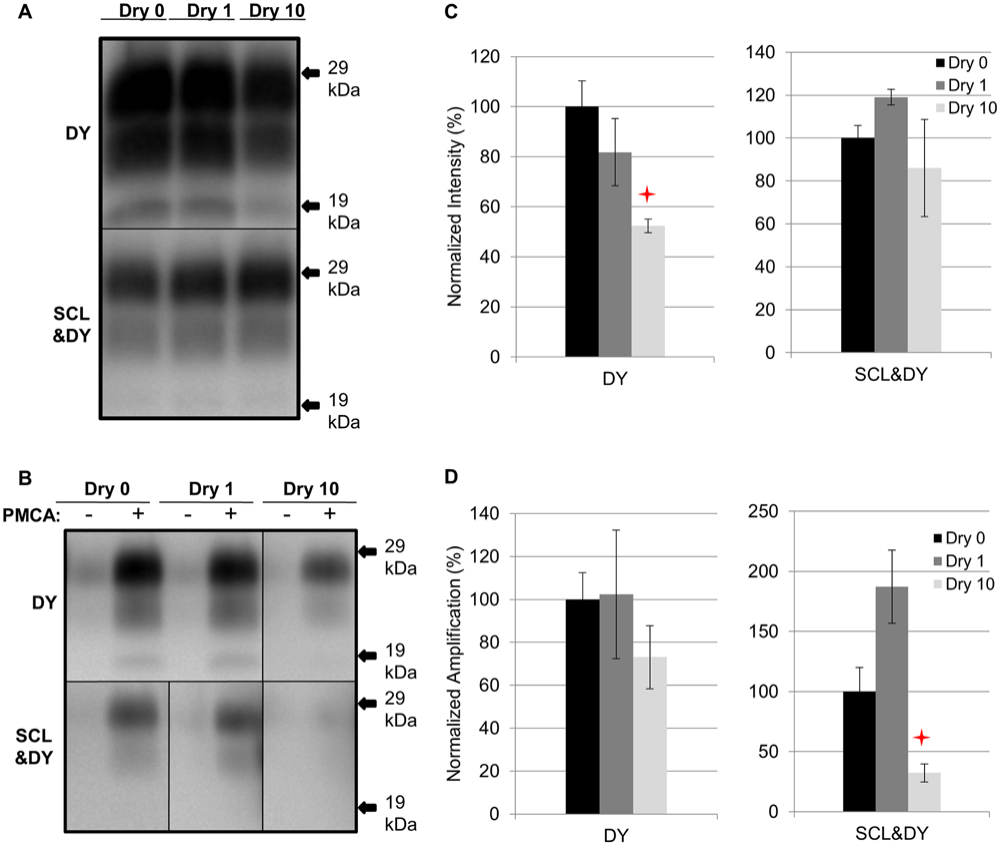
Influence of repeated cycles of drying and wetting on proteinase K resistance and amplification efficiency of DY TME. Western blot (A and B) and quantification (C and D) of PK digested and PMCA amplification (3 rounds) of DY PrP^Sc^ alone or adsorbed to SCL before (Dry 0) and after 1 (Dry 1) and 10 (Dry 10) serial rounds of drying and wetting. Negative PMCA samples were diluted from corresponding PMCA seeding with a dilution factor of 80. Migration of 29 and 19 kDa molecular weight marker is indicated on the right of the Western blot. Star indicates significant difference (p<0.05; n = 3) between treated and untreated sample.

Samples were prepared as described in Table 1. A significant (p>0.05) difference in PrP^Sc^ immunoreactivity was not observed for unbound DY or SCL-adsorbed DY after 1 drying and wetting cycle compared to the control (Fig. 4A and 4C). A significant (p<0.05) reduction in PrP^Sc^ abundance of 48% was observed for unbound DY treated with 10 cycles of drying and wetting while the PrP^Sc^ abundance of SCL-bound DY was not significantly (p>0.05) changed (Fig. 4A and 4C). After 3 rounds of PMCA, DY and SCL-bound DY amplified to similar (p>0.05) levels after 1 cycle of drying and wetting compared to controls (Fig. 4B and 4D). When exposed to 10 drying/wetting cycles, a significant reduction (p<0.05) in amplification was only observed for SCL-bound DY by 68% and not for unbound DY (Fig. 4B and 4D).

### Influence of repeated cycles of drying and wetting on proteinase K resistance and amplification efficiency of CWD

**Fig 5:**
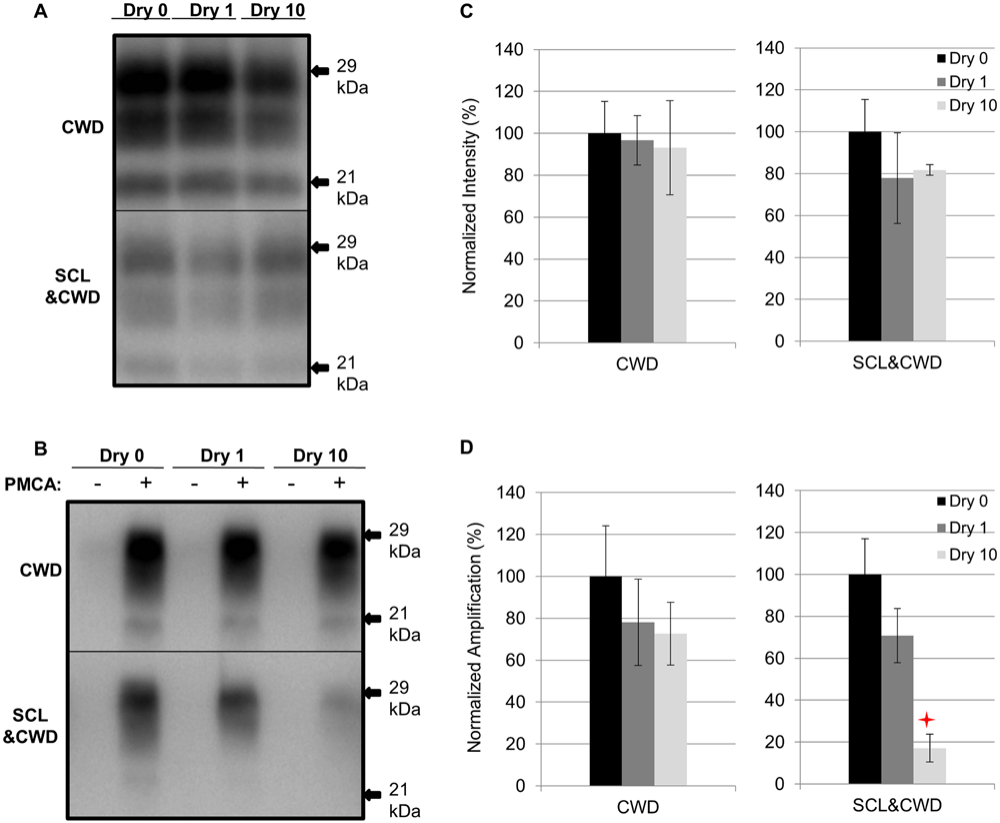
Influence of repeated cycles of drying and wetting on proteinase K resistance and amplification efficiency of elk CWD. Western blot (A and B) and quantification (C and D) of PK digested and PMCA amplification (3 rounds) of elk CWD PrP^Sc^ alone or adsorbed to SCL before (Dry 0) and after 1 (Dry 1) and 10 (Dry 10) serial rounds of drying and wetting. Migration of 29 and 21 kDa molecular weight marker is indicated on the right of the Western blot. Star indicates significant difference (p<0.05; n = 3) between treated and untreated sample.

SCL-adsorbed CWD was prepared as described in Table 1. Compared to the untreated samples (Dry 0), the PrP^Sc^ abundance did not significantly (p>0.05) differ between SCL-bound and unbound CWD with up to 10 cycles of drying and wetting (Fig. 5A and 5C). The PMCA conversion efficiency of unbound CWD after 3 rounds of PMCA was not significantly (p>0.05) different through 10 repeated cycles of drying and wetting compared to controls (Fig. 5B and 5D). In contrast, a significant (p<0.05) reduction of 83% in PMCA conversion efficiency of SCL-bound CWD after 10 cycles of drying and wetting treatment was observed (Fig. 5B and 5D).

### Sorption of HY to soil reduces the number of wet dry cycles that result in a decrease in PMCA conversion activity

**Fig 6:**
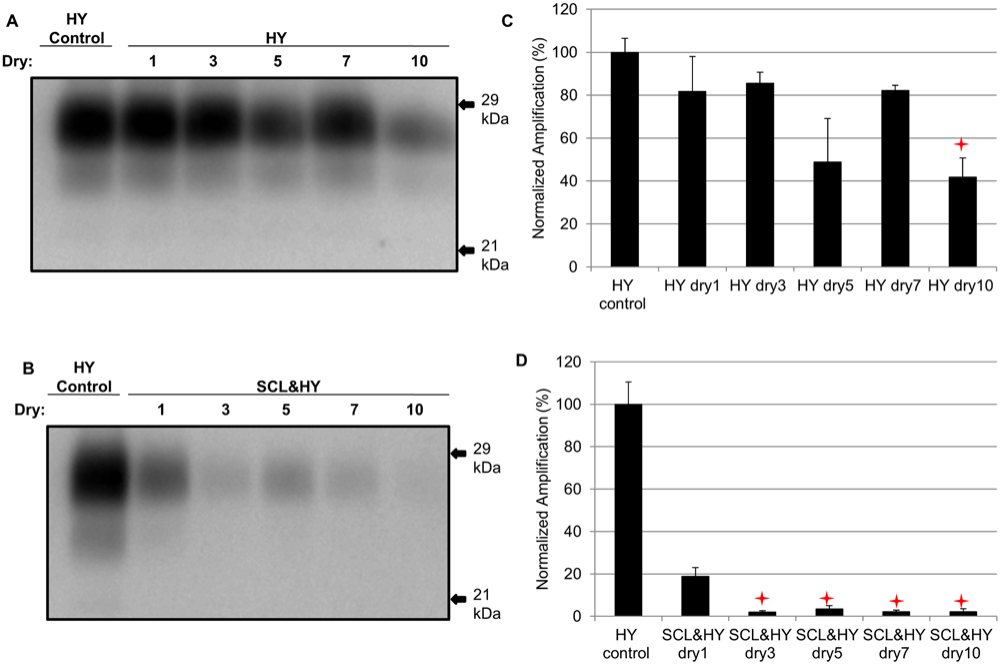
Sorption of HY to soil reduces the number of wet dry cycles that result in a decrease in PMCA conversion activity. Western blot (A and B) and quantification (C and D) of PMCA amplification (1 round) of HY PrP^Sc^ alone or adsorbed to SCL after 1, 3, 5, 7, and 10 serial rounds of drying and wetting. Migration of 29 and 21 kDa molecular weight marker is indicated on the right of the Western blot. Star indicates significant difference (p<0.05; n = 3) between multiple drying/wetting treated and single drying/wetting treated sample.

Unbound HY or SCL-adsorbed HY were subjected to 3, 5 or 7 repeated rounds of drying and wetting. Samples were then subjected to 1 round of PMCA and the abundance of amplified PrP^Sc^ was quantified. Unbound HY had similar (p>0.05) conversion efficiency at 1, 3, 5 and 7 repeated cycles of drying and wetting. However, 10 cycles of repeated drying and wetting resulted in a significant (p<0.05) reduction in HY PrP^Sc^ amplification compared to the sample treated with 1 cycle of drying and wetting (Fig. 6A and 6C). In contrast, after 3 repeated rounds of drying and wetting of SCL-bound HY amplification was significantly (p<0.05) inhibited (Fig. 6B and 6D). These results demonstrate that, under the conditions tested, binding to SCL enhances the reduction in conversion efficiency induced by repeated cycles of drying and wetting.

### PMCA conversion efficiency is decreased by sorption to soil

**Fig 7:**
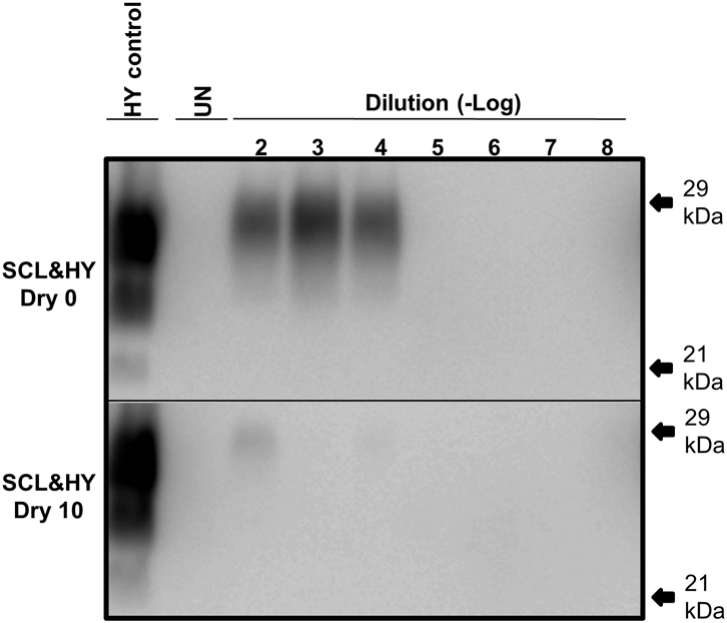
Reduced PMCA conversion coefficient of SCL-HY after repeated cycles of drying and wetting. Western blot of PMCA amplification of 10-fold serial dilutions of standardized SCL-HY PrP^Sc^ before (Dry 0) or after 10 (Dry 10) rounds of drying and wetting. Samples in replicates (n = 3) were tested. Migration of 29 and 21 kDa molecular weight marker is indicated on the right of the Western blot.

Unabsorbed and SCL-adsorbed HY was subject to 0 or 10 serial rounds of wetting and drying. The samples were adjusted to equalize the abundance of PrP^Sc^ between the samples. Ten-fold serial dilutions, ranging from 10^-2^ to 10^-8^, of these samples were subject to one round of PMCA. PMCA reactions seeded with untreated HY-SCL resulted in detectable PrP^Sc^ though the 10^-4^ dilution, while PMCA reaction seeded with an equal amount of HY-SLC PrP^Sc^ that was treated with 10 serial rounds of wetting and drying resulted in detectable PrP^Sc^ though the 10^-2^ dilution (Fig. 7). These results indicate that 10 serial rounds of wetting and drying reduce the specific activity of SCL absorbed HY PrP^Sc^ by two logs.

### Repeated cycles of drying and wetting extend the incubation period of prion infection

**Fig 8:**
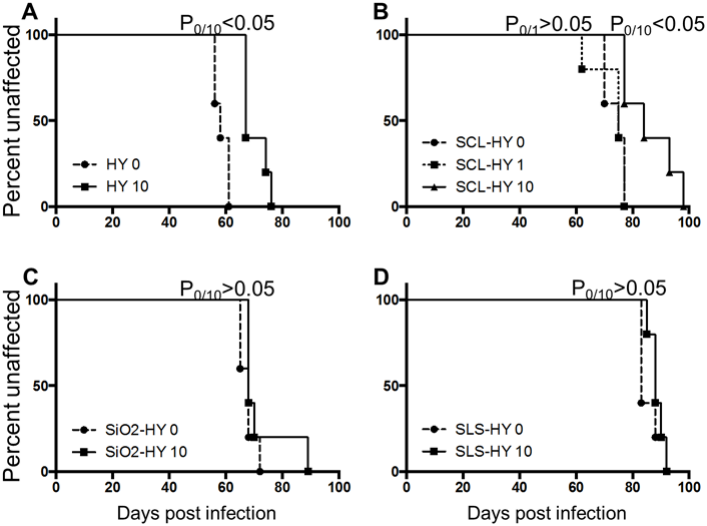
Repeated cycles of drying and wetting extend the incubation period of prion infection. Survival curve of hamsters inoculated with untreated (0) or drying/wetting treated (1 or 10) HY (A), SCL-HY (B), SiO_2-_HY (C), and SLS-HY (D). 5 hamsters (n = 5) were used for each sample. P0/1 or P0/10 designated p value derived from the comparison of incubation periods of hamsters inoculated with untreated (0) and 1 or 10 cycle(s) of drying and wetting treated samples.

Selected drying and wetting treated samples and untreated controls were intracerebrally inoculated into Syrian hamsters. Incubation periods for hamsters inoculated with HY, SCL-, SiO_2-_, and SLS-bound HY subjected to 0, 1, or 10 drying/wetting cycles are summarized in Table 2 and the survival results are presented in Fig. 8. Consistent with PK-resistance and PMCA results (Fig. 2 and 3), the incubation period of hamsters inoculated with SCL-HY subjected to 1 cycle of drying and wetting did not significantly (p>0.05) differ compared to hamsters inoculated with untreated SCL-HY (Fig. 8 and Table 2). The incubation period of HY- and SCL-HY-inoculated hamsters subjected to 10 cycles of drying/wetting was significantly (p<0.05) extended 13 days compared to that of hamsters inoculated with the untreated control (Fig. 8 and Table 2). This extension of the incubation period is consistent with a 2 log reduction in prion titer [21]. The incubation period of hamsters inoculated with SLS-HY and SiO_2-_HY treated with 10 cycles did not significantly (p>0.05) differ compared to hamsters inoculated with untreated control (Fig. 8 and Table 2).

**Table 2:** Incubation periods of hamsters intracerebrally inoculated with unbound and soil-bound PrP^Sc^.

**Inoculum**	**No. of drying/wetting cycles**	**Incubation period (days±SEM)**	**No. of hamsters affected/total number inoculated**	**P value**
HY TME	0	58±3	5/5	n.a
HY TME	10	70±4	5/5	<0.05
SCL-HY TME	0	74±4	5/5	n.a.
SCL-HY TME	1	73±6	5/5	>0.05
SCL-HY TME	10	86±9	5/5	<0.05
SiO_2-_HY TME	0	67±3	5/5	n.a.
SiO_2-_HY TME	10	69±3	5/5	>0.05
SLS-HY TME	0	86±4	5/5	n.a.
SLS-HY TME	10	89±3	5/5	>0.05

## Discussion

Prions have been detected in the environment [2–5, 44, 45] and they can survive for years in soils [2–5]. Prion shedding and persistence in the environment is well documented, however, little is known about environmental degradation of prions. In this study we investigated if prion infectivity is mitigated by natural environmental conditions. Under natural conditions of repeated wetting and drying we found evidence of PrP^Sc^ degradation, decreased PrP^Sc^ conversion activity, and an increase in prion incubation period suggesting reduced prion infectivity. This effect was dependent on the soil type that was bound to PrP^Sc^ and the source of prions, underscoring the complexity of the interaction of prions with soil.

### Reduced PrP^Sc^ abundance after repeated cycles of drying and wetting

Repeated cycles of drying and wetting degraded not only PrP^Sc^ but also other proteins in the brain homogenate whereas binding to soil prior to repeated cycles of wetting and drying eliminated this effect. (Fig. 1). While the exact mechanism of prion protein degradation due to repeated cycles of drying and wetting are not known, several possibilities exist. We hypothesize that exposure to repeated cycles of drying and wetting results in protein conformational changes that render PrP^Sc^ more susceptible to degradation. Loss of water and changes in ion concentrations and pH in solution may occur during dehydration which can affect the secondary structure of proteins [43, 46–48], however, changes in ionic strength or pH were not observed between cycles in this study. Although poorly understood, changes in soil properties such as surface charge or cation exchange capacity occur after repeated drying and wetting cycles [49, 50] that can result in desorption and/or reorganization of adsorbed compounds including proteins.

Alternatively, consolidation of the bonding between soil and the adsorbed PrP^Sc^ after drying may make PrP^Sc^ desorption more difficult resulting in reduced PrP^Sc^ detection and prion infectivity. The implication of this possibility is that PrP^Sc^ desorption is required for prion conversion and western blot detection. This explanation is consistent with previous findings that PrP^Sc^ attached to stainless steel surfaces (dried onto surface or as incubation solution) becomes more resistant to decontamination when exposed to extended drying compared with no drying or wet storage condition [51, 52]. We speculate more compact stacking of PrP^Sc^ aggregates on the surface may occur during drying as water molecules evaporate, minimizing PrP^Sc^ exposure to the surroundings.

### Repeated drying and wetting renders soil-bound prion less infectious

The reduced PMCA conversion coefficient in combination with the extended incubation period in hamster bioassay compared to untreated prions (Fig. 3 and 8, Table 2) are consistent with a 2 log reduction in prion infectivity of soil-bound prions [21] after 10 cycles of drying/wetting. While both PMCA and bioassay data are in agreement with a 2 log reduction in titer following 10 cycles of drying/wetting, since titer was not directly calculated, this value should be interpreted with caution. It is unknown if additional cycles of wetting will further reduce infectivity or if complete prion inactivation is possible. The observed reduction in infectivity is not entirely due to loss of PrP^Sc^. When standardized for the amount of starting PrP^Sc^, the PMCA conversion coefficient of HY bound to SCL that has not been treated to repeated cycles of wetting and drying is two orders of magnitude greater compared to HY-SCL that has been repeatedly wetted and dried for 10 cycles (Fig. 6). Since the reduction in the specific activity of PrP^Sc^ is enhanced when HY is bound to SCL, we hypothesize at the PrP^Sc^-SCL interface, the wetting and drying process is altering a property of PrP^Sc^ that renders it less infectious.

### Effect of repeated drying and wetting on the properties of soil-bound prions is soil type and strain dependent

Changes in PrP^Sc^ abundance and PMCA conversion efficiency after repeated cycles of drying and wetting are observed for a subset of unbound and soil-bound PrP^Sc^. HY and DY PrP^Sc^ are more susceptible to PK digestion following repeated cycles of drying and wetting compared to CWD (Figs. 2, 3, 4, and 5). Binding of DY PrP^Sc^ to SCL protects DY PrP^Sc^ by enhancing PK resistance but results in a reduction in DY and CWD PrP^Sc^ conversion activity (Figs. 4 and 5). Adsorption of the same PrP^Sc^ to different soils resulted in a greater or lesser effect of repeated drying/wetting on PrP^Sc^ properties (Figs. 2 and 3). Factors that may contribute to the variation include soil surface properties and soil-prion bonding. Since the percentage of sand and clay in a soil resulted in different soil stability changes in response to drying/wetting cycles we hypothesize that clay-clay and clay-sand interactions can indirectly affect the soil-prion interface [41]. Overall, this dynamic can affect which prion strains persist and the relative titer in any given environment. Since the relative ratios of prion strains can affect strain emergence, the environment would be expected to have a strong influence on the overall dynamics of transmission and strain emergence.

### Environmental factors affecting the efficiency of repeated drying and wetting

Since prions are likely to be immobile in surface soils [26], soil-bound prions are readily accessible to ambient weather conditions which can include changes in surface soil moisture. In some CWD-endemic areas such as Phantom Valley (Latitude: 40.4°N; Longitude: 105.9°W) close to Rocky Mountain National Park, the number of moisture change in surface soil (soil depth is 0.5 m) can be up to 28 per month [53]. Based on our findings, drying/wetting cycles which repeatedly change soil moisture may be a natural degradation pathway for soil-bound prions. Additionally, wetting and drying can change microbial activity in soils [39, 40] which may affect prion-soil interactions. Ambient temperature likely does not contribute to natural prion degradation since most heat induced decontamination (incomplete inactivation) occurs at temperatures well over 100°C [54–57]. Enzymes secreted from soil microorganisms, such as serine proteases, may be auxiliary for soil-bound prion degradation and inactivation, however, they were only found to be effective at high temperature, high pH or both [58, 59]. Overall, this study provides the first evidence that natural processes can reduce prion infectivity. Since the total environmental prion load is a balance between addition of prions to the environment and clearance of prions from the environment, efforts to limit prion input into the environment may positively affect this balance and have meaningful results in reducing environmental transmission of prion diseases. Additionally, the soil composition and hydrology of an area may shape the overall transmission dynamics and alter strain prevalence of prions.

## Materials and Methods

### Prion sources and tissue preparation

Prion-infected brain tissues were collected from hamsters infected with either the hyper (HY) or drowsy (DY) strain of transmissible mink encephalopathy (TME) or from a naturally infected elk with CWD agent as described previously [20]. Infected brains were homogenized to 10% (w/v) in Dulbecco’s phosphate-buffered saline (DPBS) without Ca^2+^ or Mg^2+^ (Mediatech, Herndon, VA) using strain-dedicated Tenbroeck tissue grinders (Kontes, Vineland, NJ). Uninfected Syrian hamster and uninfected Tg(CerPrP)^1536^ mouse brain was homogenized to 10% (w/v) in ice-cold PMCA conversion buffer (DPBS [pH 7.5] containing 5 mM EDTA, 1% (v/v) Triton X-100, and a complete protease inhibitor tablet [Roche Diagnostics, Mannheim, Germany]). The homogenate was centrifuged at 460×g for 30 seconds and the supernatant was collected and stored at -80°C.

### Prion adsorption to soils and soil minerals

Soils and soil minerals used in this study included sterile Rindasilty clay loam (SCL) soil (a VerticEpiaqualf); sterile Dickinson sandy loam soil (SLS, a TypicHapludoll); sodium bentonite clay (CETCO, Arlington Heights, IL); silicon dioxide powder (Sigma Aldrich, St. Louis, MO); humic acid (HA)-coated silica gel particles (SiO_2_ HA); and gamma-irradiated fine white sand (Fisher Scientific, Pittsburgh, PA). Physicochemical properties of these soils and soil minerals have been described previously [19, 38]. To obtain soil bound prions, 10% brain homogenate (BH) was added to soil and for each soil or soil mineral, the incubation time and prion to soil ratios, were selected based on previous studies [19, 20] (Table 1). Each BH-soil combination was prepared in triplicate. The BH-soil mixture was rotated at 24 rpm (Mini Labroller, Edison, NJ) at room temperature. Samples were removed after incubation and centrifuged at 100× g for 5min. The supernatant was removed and the pellets were washed a minimum of three times with 1× DPBS. The soil pellets were resuspended in 1× DPBS at concentrations described in Table 1 and were stored at -80°C until use. HY TME, DY TME, and elk CWD BH were used as unbound controls.

### Drying and wetting treatment

Each sample was placed in an uncapped 200 µL PCR tube (Thermo Scientific) and incubated at 40°C. Samples were dried and weighed periodically until there was less than a 0.5% change in weight resulting in a minimum of 7 hr drying time. To perform consistently, around 12 hours’ drying is selected for one cycle. Dried samples were rehydrated with 10 µl of ultrafiltered deionized water and mixed thoroughly. The drying followed by rewetting constituted one drying/wetting cycle. Conductivity and pH were measured with an Oakton 700 bench top meter using a 3-point calibration curve. After the desired number of treatment cycles, samples were stored at -80°C until use.

### Animal bioassay

Intracerebral (i.c.) inoculations of Syrian hamsters (Harlan Sprague-Dawley, Indianapolis, IN) were conducted as described previously [30, 60]. Silty clay loam soil HY TME (untreated, and 1- and 10-drying/wetting-cycle treated) and silicon dioxide powder adsorbed HY TME (untreated, and 10-drying/wetting-cycle treated) were selected as inocula. The incubation period was determined as the length of time in days between inoculation and the onset of clinical signs of HY TME.

### PMCA

Protein misfolding cyclic amplification (PMCA) was performed as described previously [61]. Sonication was performed with a Misonix (Farmingdale, NY) 4000 sonicator with amplitude set to level 75, generating an average output of 160 W during sonication treatment. Samples were diluted with 10% (w/v) uninfected hamster or elk brain homogenate at 1:100 for HY TME and CWD, and 1:20 for DY TME for the first round. After one round, homogenate from round 1 was diluted at 1:20 for HY TME, 1:10 for elk CWD, and 1:1 for DY TME for the subsequent rounds. Each round was performed at 37°C for either 24 hr for HY TME and DY TME consisted of 144 cycles of 5 s sonication followed by 9min 55s of incubation or 48 hr for elk CWD consisted of 288 cycles with the same sonication/incubation time as HY TME and DY TME. Before each PMCA round, an aliquot was placed at -80°C as an unsonicated control. Samples containing only 10% (w/v) uninfected brain homogenate were included with each PMCA round as negative controls.

### Western blot analysis

Western blot analysis was performed as described previously [62]. Briefly, samples were incubated and digested with 22.5 µg/ml (HY TME/DY TME) or 45 µg/ml (elk CWD) proteinase K (PK) (Roche Diagnostics Corporation, Indianapolis, IN) at 37°C for 30 min (HY TME/DY TME) or 1 hr (CWD) with constant agitation. The PK digestion was terminated by boiling in 1x SDS-PAGE sample buffer (final concentration). The samples were size fractionated with 12.5% SDS-PAGE and transferred to a polyvinylidenedifluoride membrane (NuPage; Invitrogen, Carlsbad, CA). The membrane was blocked with 5% w/v nonfat dry milk in 1× TTBS (Bio-Rad Laboratories, Hercules, CA) for 30 min. Hamster samples were immunoblotted with MAb 3F4 (Chemicon, Temecula, CA; 1:10,000). Elk/Tg(CerPrP)^1536^ samples were immunoblotted with 8H4 (1:10,000). The blots were developed with Supersignal West Femto maximum sensitivity substrate, according to the manufacturer’s instructions (Pierce, Rockford, IL), imaged on a 4000R imaging station (Kodak, Rochester, NY), and analyzed using Kodak (New Haven, CT) molecular imaging software, V.5.0.1.27. Densities of sample replicate (n≥3) intensities were standardized to brain homogenate controls on the same gel to control for inter-gel variance. PMCA amplification is determined as the absolute difference of intensity density between unamplified and sonicator-amplified samples. For each type of soil, unbound prions and untreated samples (Dry 0) were used as controls to determine the effect of drying/wetting (Dry 1 and Dry 10) on PrP^Sc^ seeding efficiency. Intensities of the same control were averaged out through the entire study. Statistical analysis (Student’s *t* test with Welch’s correction, two-tailed P value) was performed using Prism 6.0 (GraphPad Software, Inc., San Diego, CA) by separately comparing samples with each treatment to the untreated control or samples with the least treatment.

### SYPRO Ruby gel staining

Separated proteins in the polyacrylamide gel were stained with SUPRO Ruby following the protocol provided by the manufacturer. Briefly, the gel was placed in a clean plastic dish and incubated in a fixative solution containing 10% methanol and 7% acetic acid at room temperature with gentle agitation for two 15 minutes. Then the gel was incubated in undiluted SYPRO Ruby staining solution overnight without being exposed to light. Stained gel was then transferred to a clean plastic dish and washed with 10% methanol and 7% acetic solution for two times followed by one time rinse with MQ H_2_O. The gel was imaged in an electrophoresis gel imaging imager cabinet (Bio-rad Universal Hood ii) using UV epi-illumination and analyzed by a 1-D analysis software Quantity One version 4.6.7. The lightness density of samples of interest were compared and significance was analyzed with build-in *t* test (two-tailed p value with unequal variance) in Microsoft Excel 2013.

### Ethics statement

All procedures involving animals were approved by Creighton University Institutional Animal Care and Use Committee (protocol number 0 872 and 0881) and comply with the Guide for the Care and Use of Laboratory Animals.
